# Porcelain Inlays from the Clinical Standpoint—Cavity Preparation

**Published:** 1905-09

**Authors:** James G. Palmer

**Affiliations:** New York


### PORCELAIN INLAYS FROM THE CLINICAL STANDPOINT—CAVITY PREPARATION.[1]

BY JAMES G. PALMER, NEW YORK.

I HAVE prepared a few cavities in some natural teeth which will be passed around for your inspection. These cavities are the most common types of those in which I have inserted porcelain. My work in this direction does not seem to me to have been extensive enough to warrant your committee in their request that I speak upon this subject, and the more intricate cavities, especially restoring contour in the incisors, must be left for those who are better acquainted with such work than I am.

In our ordinary every day work on the labial or buccal surfaces of the teeth, and in approximal cavities of ordinary character and size—at any rate, cavities not of a complicated character—the first step, of course, is the removal of all decay which is to be accomplished with burs or excavators as the operator pleases.

After the removal of this *débris,* the cavity is shaped with *inverted-cone* burs or square-end fissure burs so that the walls shall be as nearly parallel as possible. Burs of these shapes, with square or flat ends, will form a nearly level floor, within the cavity, of quite a uniform depth, and make the angles at the bottom of the cavity fairly well defined. The edges of the cavity should be shaped in graceful curves rather than irregular outlines. Many a large contour gold-filling has been made less conspicuous by proper attention to these curves—to the "line of beauty," if I may use the term—especially on the labial aspect of the tooth, than similar fillings, when attention has not been given to this little detail.

This same principle, I think, applies in some degree to porcelain inlays, and for that reason I say avoid irregular outlines. I have also been impressed with the fact that greater care should be given to preparing the floor of the cavity than has usually been done. If this floor is nearly level and the cavity as nearly as may be of a uniform depth, the inlay will be much more readily retained. In this I find the square or flat end of the

---

[1] Read before The New York Institute of Stomatology, May, 1905.

inverted cone, the fissure bur, and the square-edge wheel bur of great value.

Having thus formed the cavity *in the rough,* as it might be called,—being especially careful, if the cavities are small, that they be deep enough to give sufficient thickness to the inlay so that the "shadow problem" shall be eliminated,—I have used, to finish the edges of the cavities, almost entirely, the fine-cut *plug-finishing* burs.

So much of my work has been in cavities of small size that I have not been able to use Arkansas, Scotch, or other stones to my satisfaction. With a peculiar barrel-shaped plug-finishing bur, having different sizes at hand, I have been able to shape the edges of the cavities quite as I desired. I have also used in connection with these burs a large wheel *plug-finishing* bur with a flat, instead of rounded circumference. When new these burs cut surprisingly well, and with them the walls of the cavity can readily be paralleled. Sometimes it may be desirable to use a stone or sand-paper disk to smooth the tooth itself, so that, with this smoothing or polishing on the outside of the cavity, on the surface of the tooth, and close attention to smoothing the edges of the cavity within, the outlines of the cavity will be almost a knife-edge, so sharply will they be defined. I have always endeavored to avoid sharp or square angles within the cavity or on the line of the circumference, preferring to *round* these corners, if that is understandable.

Where cavities extend underneath the gum, a wisp of cotton tightly rolled and saturated with *adrenalin chloride,* has proved in my hands of great value. This cotton can be tucked well under the gum, even to the point of drawing blood, which the adrenalin promptly checks, and if the mouth and lips have been protected with cotton rolls and napkins, one can frequently complete the entire operation without using the dam.

In some of the larger cavities, by reason of the extent of decay or the shape of the cavity, it may be impossible to adhere to the rule concerning parallel walls. Indeed, I think more stress has often been placed upon paralleling than need be. In some of these large cavities a very long bevel may advantageously be made, with a square floor, something like a cork.

The square end and *long* bevel gives a strong hold when inserted.

In addition to the plug-finishing burs of which I have spoken, the very heavy and *very sharp* chisel is often of great value, especially in the larger cavities.

In very large, but shallow, cavities or restorations on the labial aspect of the six front teeth, where abrasion or erosion has extended a great distance toward the incisal edges, and entirely across the face of the tooth, it is frequently impossible to deepen the cavity on either approximal aspect as much as would seem desirable for purposes of retention. In such cases, however, the wall at the gum line and its opposite toward the incisive portion of the tooth may be made nearly, if not quite, parallel, and will be found quite sufficient to retain such inlays if they fit otherwise.

In large contour work on molars or bicuspids, it has always been my aim to have the floor of the cavity as nearly square or at right angle to the walls as possible, and to cut back the edges sufficiently to make them nearly straight, so that the stress of mastication may be directly through the tooth rather than across it.

I have here one model of a cavity in a devitalized right superior first molar. This cavity is on the anterior aproximal and coronal portion of the tooth, and is presented as a practical case. The inlay was made to-day in my office. You will observe that the floor of the cavity is nearly at right angles to the walls. Of course, such a marked square shape could not be obtained in a live tooth.

